# Combined radiotherapy and immune checkpoint inhibition for the treatment of advanced hepatocellular carcinoma

**DOI:** 10.3389/fonc.2023.1193762

**Published:** 2023-07-24

**Authors:** Alexander H. Shannon, Ashish Manne, Dayssy A. Diaz Pardo, Timothy M. Pawlik

**Affiliations:** ^1^ Department of Surgery, Division of Surgical Oncology, The Ohio State University Wexner Medical Center, Columbus, OH, United States; ^2^ Department of Internal Medicine, Division of Medical Oncology at the Arthur G. James Cancer Hospital and Richard J. Solove Research Institute, The Ohio State University Comprehensive Cancer Center, Columbus, OH, United States; ^3^ Department of Radiation Oncology, The Ohio State University, Comprehensive Cancer Center-James Hospital and Solove Research Institute, Columbus, OH, United States

**Keywords:** radiotherapy, immune checkpoint inhibitors, hepatocellular carcinoma, combination therapy, stereotactic body radiotherapy

## Abstract

Hepatocellular Carcinoma (HCC) is one of the most common cancers and a leading cause of cancer related death worldwide. Until recently, systemic therapy for advanced HCC, defined as Barcelona Clinic Liver Cancer (BCLC) stage B or C, was limited and ineffective in terms of long-term survival. However, over the past decade, immune check point inhibitors (ICI) combinations have emerged as a potential therapeutic option for patients with nonresectable disease. ICI modulate the tumor microenvironment to prevent progression of the tumor. Radiotherapy is a crucial tool in treating unresectable HCC and may enhance the efficacy of ICI by manipulating the tumor microenvironment and decreasing tumor resistance to certain therapies. We herein review developments in the field of ICI combined with radiotherapy for the treatment of HCC, as well as look at challenges associated with these treatment modalities, and review future directions of combination therapy.

## Introduction

Hepatocellular Carcinoma (HCC) is one of the most common cancers and a leading cause of cancer-related death worldwide (1). Risk factors for HCC include hepatitis B virus (HBV), hepatitis C virus (HCV), non-alcoholic fatty liver disease (NAFLD), alcoholic cirrhosis, tobacco use, and inherited disorders such as hemochromatosis, Wilson’s disease, and alpha-1 antitrypsin deficiency (2). Treatment options for HCC are various and depend on extent of tumor burden, underlying liver disease, and performance status. Options for treatment include resection, transplantation, locoregional and systemic therapies. Given various treatment options, a multi-disciplinary approach to care is essential, with surgical resection or transplant offering the best chance for cure. Unfortunately, many patients are not eligible for surgery given the advanced stage of disease at diagnosis. Consequently, HCC has a poor prognosis with five-year survival of 20-40% (3, 4).

Until recently, systemic therapy for advanced HCC was limited to tyrosine-kinase inhibitors (TKI) and ramucirumab (for AFP > 400ng/ml) and were ineffective in improving long-term survival. First line systemic therapy consisted of sorafenib, a tyrosine kinase inhibitor (5). However, over the past decade, immune check point inhibitors (ICI) combinations have emerged as a potential therapeutic option for patients with advanced stage disease, defined as Barcelona Clinic Liver Cancer (BCLC) stage B or C. ICI have a proven benefit in a multitude of other malignancies such as melanoma, breast, and colon cancer among others, but only recently has been this success been extrapolated to HCC (6–8). The IMbrave 150 trial demonstrated that, compared to sorafenib, the combination of atezolizumab (PD-L1 inhibitor) and bevacizumab (VEGF inhibitor) had improved overall (OS) and progression free survival (PFS) in patients with unresectable HCC (9). Additionally, the CheckMate 040 trial showed promise in the use of nivolumab (PD-1 inhibitor) and ipilimumab (anti-CTLA-4) with combination therapy having favorable objective response rates (ORR) among patients who had previously been treated with sorafenib (10).

The tumor microenvironment and its interaction with host immune cells plays an integral role in preventing cancer progression. Cirrhosis and chronic inflammation from viral hepatitis, alcohol or NAFLD can lead to changes in the hepatic immune response that favor carcinogenesis, which is part of the pathophysiology of HCC. ICI modulate the tumor microenvironment to prevent progression of tumor disease. Immunotherapy has demonstrated improvement in survival after chemoradiation, and it is now considered standard of care in certain cancers, such as non-small cell lung cancer (11). Recently, radiotherapy, in addition to ICI, has demonstrated potential in the treatment of advanced HCC. Radiotherapy is a crucial tool in the treatment of unresectable HCC, and the combination of radiotherapy with sorafenib has demonstrated improved OS and PFS (12). Radiotherapy may enhance the efficacy of ICI by manipulating the tumor microenvironment and decreasing tumor resistance to certain therapies (13–15). Given this, the objective of the current review is to highlight developments in the field of ICI combined with radiotherapy for the treatment of HCC, characterize challenges with these treatment modalities, as well as highlight future directions of combination therapy.

## Methods

**Figure 1:**
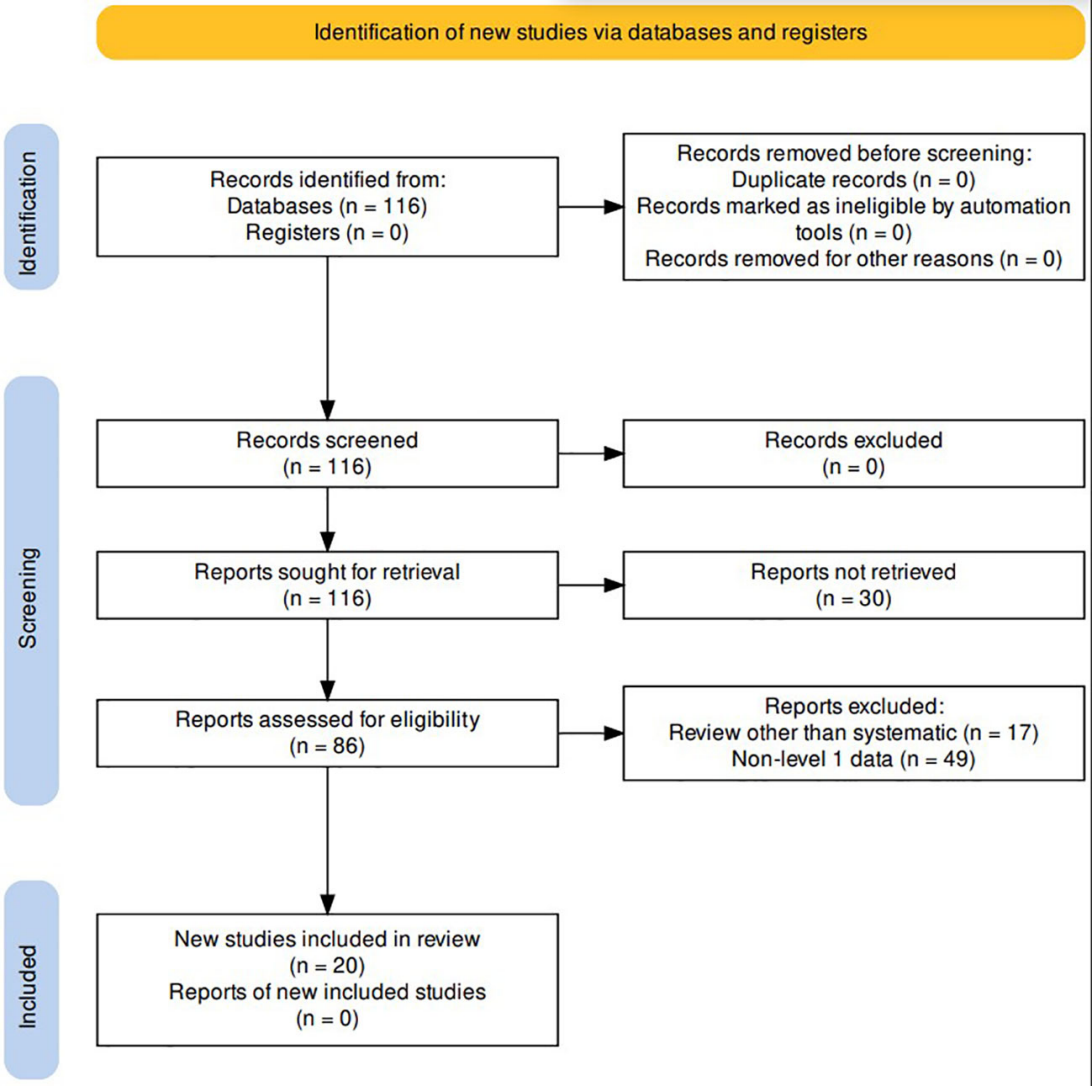
PRISMA Flow Diagram.

A comprehensive review of the literature was conducted in the PubMed database for studies published between January 2010 through February 2023. The following keywords and MESH terms were included in the search: “radiotherapy,” “immune checkpoint inhibitor,” “hepatocellular carcinoma.” Records were excluded if not written in English or if the full report was not available; reviews that were not systematic in nature (n=17), as well as reports that did not include level 1 data (n=49) were also excluded. A total of 20 studies were included in the final analysis (**Figure 1**).

## Immune checkpoint inhibition for HCC

ICI target inhibitory and stimulatory immunoreceptors, as well as act as regulators of the immune system. ICI therapy has less systemic side effects than cytotoxic chemotherapy with may have more durable responses compared with targeted therapies (16). Tumors cells are unique in their ability to downregulate stimulatory immunoreceptors while upregulating inhibitory immunoreceptors, thereby evading host immune cells and allowing propagation of malignant cells (17). The tumor microenvironment contributes to the suppression of many host innate and adaptive immune cells (18). Among the most prominent and well-studied immunoreceptors responsible for this evasion are PD-1 and PD-L1 and CTLA-4 (19–21). By blocking these immunoreceptors, ICI enhance the antitumor function of host immune cells to curb the spread of malignant cells (16).

Recent trials have demonstrated the efficacy of ICI compared to systemic cytotoxic chemotherapy among patients with advanced HCC. Initially, single agent monotherapy was utilized for treatment; however, the CheckMate 459 study noted no improvement in OS or PFS among patient treated with nivolumab (PD-1 inhibitor) versus sorafenib (22). In turn, focus shifted to combination ICI therapy to address tumor heterogeneity. As noted, the IMbrave 150 trial was a landmark phase III multi-center global trial that compared atezolizumab plus bevacizumab (Atezo-Bev) versus sorafenib among patients with unresectable HCC. Importantly, Atezo-Bev was associated with an improved one-year OS (67.2% Atezo-Bev versus 54.6% sorafenib) and PFS (6.8 months Atezo-Bev versus 4.3 months sorafenib) (23). A recent update of the study from 2022 noted continued improvement in OS and PFS in the Atezo-Bev group (9). Consequently Atezo-Bev was recommended as first line treatment for advanced HCC according to the 2022 BCLC guidelines (5).

Other studies of ICI have investigated tremelimumab, an anti-CTLA-4 monoclonal antibody in combination with durvalumab, an anti-PD-L1 monoclonal antibody. The phase III HIMALAYA trial evaluated 1000 patients with unresectable HCC who had no prior treatment who had preserved liver function with good performance status (24). This study noted that combination therapy with tremlimumab and durvalumab was associated with improvement in ORR and OS versus sorafenib (ORR 20 *vs* 5% and OS 16.4 *vs* 13.3 months, respectively) (24). Additionally, the trial demonstrated the inferiority of durvalumab monotherapy versus sorafenib. The safety profiles were similar and main side effects were rash and transaminitis (25). Overall, the HIMALAYA trial demonstrated that tremelimumab and durvalumab may be an acceptable first line alternative to Atezo-Bev.

Another combination therapy regimen of nivolumab, a PD-1 inhibitor, and ipilimumab, another CTLA-4 inhibitor was evaluated in the CheckMate 040 trial (10). In this study, combination therapy with these two drugs was compared with nivolumab monotherapy among 148 patients who had advanced HCC and had previously been treated with sorafenib (10). Of note, there was improved ORR and OS among patients in the combination group; however, there were more side effects including hypothyroidism and adrenal insufficiency. Nivolumab monotherapy has also demonstrated to improve outcomes among patients with HCC, although its impact seemed to be augmented by the addition of ipilimumab (22). Another study assessed pembrolizumab monotherapy as second line treatment for advanced HCC among patients previously treated with sorafenib; OS, ORR, and PFS) were all improved versus placebo (26). As such, pembrolizumab, a anti PD-1 monoclonal antibody, has been approved in the United States as second line therapy for advanced HCC previously treated with sorafenib (27).

## Radiotherapy for HCC

Radiotherapy (RT), including external beam radiotherapy (EBRT) and stereotactic body radiotherapy (SBRT) and radioembolization (RE), has evolved over the years to play a crucial role in the treatment of certain cancers, including lung and rectal cancer (28, 29). RT’s role in the treatment of unresectable HCC compared to or in combination with other locoregional therapies continues to emerge, with SBRT and RE options being used in select patients with intermediate and advanced stage HCC (30, 31). Early RT techniques, such as EBRT damaged not only the HCC but also surrounding healthy liver parenchyma, leading to liver insufficiency and radiation induced hepatitis (32). Additional adverse effects from EBRT included ulcers, gastrointestinal bleeding, and pneumonitis (33). More modern techniques have allowed radiation oncologists to target more focal areas of the liver limiting damage to surrounding healthy tissue/viscera. Techniques such as SBRT, in which there is a limited number of high dose RT fractions delivered to a focused area of tumor, minimizes the amount of extraneous radiation to other healthy tissue (34). RE or selective internal radiation therapy is another recent technique that provides focused radiation via radio-labeled Yttrium-90 microspheres directly into the hepatic artery (35). HCC is a hypervascular tumor, with tumor being preferentially supplied by the hepatic arteries and normal hepatocytes receiving their blood supply from the portal vein (36). Consequently, radiation preferentially travels to the tumor via the hepatic arteries (37).

Study on SBRT has increased over the past decade. Wahl et al. compared 224 patients with unresectable HCC without metastases treated with SBRT versus radiofrequency ablation (RFA), demonstrating lower local progression in the SBRT group at one and two years and equivalent OS over the same time (38). Another phase III trial compared proton beam radiotherapy to radiofrequency ablation in patients with recurrent HCC and showed proton beam radiotherapy was non inferior in terms of 2 year PFS (39). Local control rates for advanced HCC treated with SBRT range from 68-95% three years after treatment (40–42). SBRT has been used as bridge to transplant, with comparable outcomes to RFA and Transarterial chemoembolization (TACE) (43). Data have suggested a benefit of SBRT among patients with advanced HCC with portal vein tumor thrombus or inferior vena cava tumor thrombus (44, 45). Additionally, the recent NRG/RTOG 112 phase III clinical trial, showed improved OS, PFS, and quality of life at 6 months without increase in adverse effects for patients with unresectable HCC treated with SBRT followed by sorafenib compared to sorafenib alone (12). Of note, only patients with a limited burden of extrahepatic disease were eligible for enrollment. Although the National Comprehensive Cancer Network (NCCN) includes radiation as an option for patients who are not eligible for transplant there is still a paucity of data directly comparing SBRT to other locoregional therapies (37, 46). SBRT is contraindicated in patients without adequate residual normal liver volume outside the radiation field and in patients with Child-Pugh class B and C cirrhosis (47).

RE has also been studied among patients with advanced HCC. In fact, several randomized trials have compared RE versus sorafenib. Neither The SIRveNIB nor the SARAH trial showed improvement is OS, however the SIRveNIB trial demonstrated superiority of RE over sorafenib in terms of PFS and time to progression, although the SARAH trial noted no difference in PFS (48, 49). Additionally, a different recent randomized controlled trial noted that RE and sorafenib was not associated with improvement in OS versus sorafenib alone (50). The STOP-HCC trial is an ongoing phase III clinical trial that is investigating RE plus sorafenib versus sorafenib alone, which is still enrolling patients (51).

## Challenges to monotherapy with ICI or radiotherapy for HCC

While ICI and RT have a proven benefit in the treatment of advanced HCC, these treatments can be associated with clinical challenges. For example, while ICI has demonstrated initial success relative to many cancer types, patients can develop resistance with use (52, 53). Although there are limited data on ICI resistance, several mechanisms for resistance have been proposed. The most studied is Beta-catenin activation secondary to mutation in CTNNB1 gene, which may lead to increased apoptosis in liver cells via nuclear factor κB and decreased recruitment of dendritic cells leading to tumorgenesis (54, 55). Other pathways of resistance include downregulation of antigen processing and presentation via HLA deletion, downregulation of cytokines and signaling pathways (example loss of JAK1/2 function), tumor infiltrating lymphocyte (TIL) exclusion via deletion of PTEN gene and VEGF upregulation, and expression of other coinhibitory checkpoint receptors (56–62). Although these mechanisms have been validated in other types of cancer such as lung and colorectal, these mechanisms have yet to be fully elucidated in HCC.

RT also can have several challenges in the treatment of HCC. For example, there are no consensus guidelines regarding use of RT for treatment of HCC, although most recent NCCN guidelines recommend consideration of EBRT or SBRT as an alternative to ablation or embolization if these therapies have failed or are contraindicated (63). While some clinicians recommend RT for patients with well compensated liver function and patients who have adequate liver volume outside radiation field, there are several relative contraindications to RT. Contraindications may include patients with Child-Pugh Class B or C.

Complications of RT vary from transient to life threatening. Common short term adverse effects include fatigue and nausea while longer term effects include sequela of hepatic injury such as ascites, transaminitis, and, thrombocytopenia (64). In certain rare cases, biliary stenosis can occur as well as radiation induced hepatitis. Additionally, radiation injury to nearby structures such as the stomach, small bowel, colon, ribs, diaphragm can also occur (65, 66). In addition, there are adverse effects associated with ICI therapy. In first line therapy of HCC, atezolizumab has been associated with adverse effects including skin rash, electrolyte abnormalities, anemia, and transamnitis; bevacizumab has been associated with hypertension, abdominal pain, and diarrhea. Serious adverse effects of acute coronary syndromes, vasculitis, and immune mediated myocarditis, as well as immune mediated rashes such as Stevens-Johnson syndrome, and toxic epidermal necrolysis may be associated with nivolumab (67–69).

## Combination of ICI and radiotherapy for HCC

Given the potential benefits of both ICI and RT alone in the treatment of HCC, combination therapy with the two modalities is actively being explored. The basis for this combination approach is a hypothetical synergistic effect, which may augment the efficacy of each treatment. RT for cancer is thought to cause irreversible damage to tumor cell DNA thereby initiating cell apoptosis (70). Additionally, the immune system’s role in controlling tumor growth is well established, as it has been demonstrated that cancer survival has been associated with T cell infiltration into the tumor, as well as increased risk of cancer developing in immunosuppression patients (71–73). However, recently, RT’s role in inducing an immune response has become an area of interest with data suggesting that RT augments the efficacy of ICI by impacting the tumor microenvironment (13). The mechanism by which this synergistic effect occurs, however has not been well elucidated.

One proposed mechanisms involves RT-induced direct tumor cell death that stimulates a tumor-specific immune response and modification of the local tumor microenvironment and increased immune cell migration into the tumor (74–76). In addition, by eliminating tumor cells, intracellular contents including antigens and damage-associated molecular patterns are released, which further induce an immune response and lymphocyte infiltration that can augmentate the effect of ICI (14, 77). In particular, CD8+ T-cells and dendritic cells seem crucial to this immune response whereas CD4+ T-cells and macrophages are not as integral (78, 79). Du et al. noted that cyclic guanosine monophosphate-adenosine monophosphate synthase (cGAS) stimulates the interferon gene (STING) pathway and is crucial in RT-induced antitumor immune responses via increased immune check point PD-L1 expression (80). In a study by Kim et al. that compared combination of RT and anti-PD-L1 immunotherapy versus RT or anti-PD-L1 alone in a murine HCC model, there was less tumor growth and longer survival. The authors noted that radiation upregulated PD-L1 expression through IFN-gamma/STAT3 signaling, which might augment the action of immunotherapy (81). In a separate study, Yoo et al. also reported that combination therapy decreased tumor size in a murine HCC model (82). A propensity score matching study compared the combination of anti-PD-1, antiangiogenic therapy and RT (n=54) versus anti-PD-1 and anti-angiogenic therapy alone (n=143) (83). The data demonstrated that the addition of RT improved ORR (42.6% *vs* 24.5%), median OS (20.1 *vs* 13.3 months), and PFS (8.7 *vs* 5.4 months) (83). Similar findings using combination therapy have been reported in other cancer types such as breast and colon cancer (84, 85). This finding has been attributed this to the abscopal effect, which is the deterioration of tumors outside the radiation field during or after RT (86).

**Figure 2:**
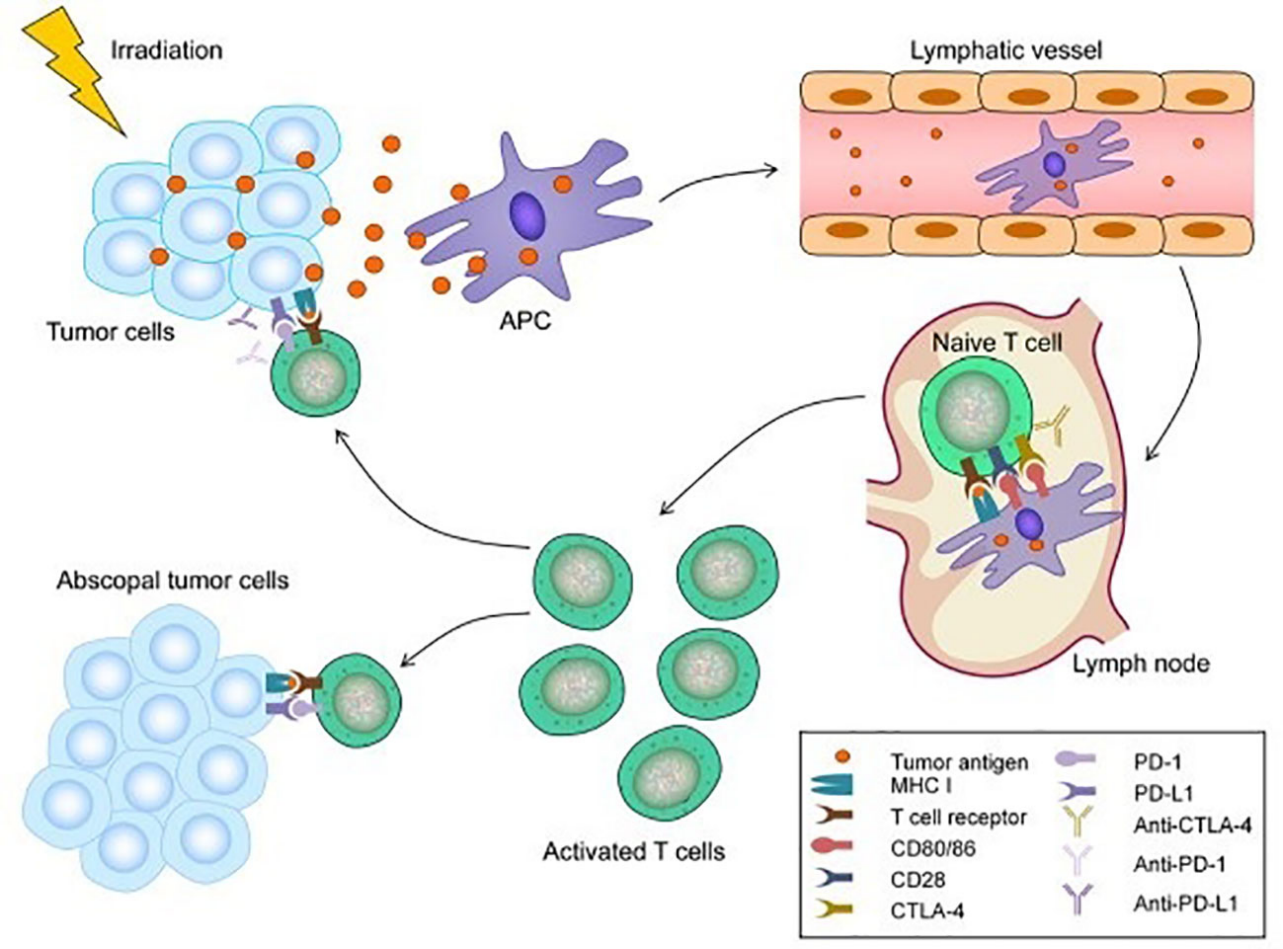
Mechanism of the abscopal effect. Radiotherapy (RT) can lead to immunogenic cell death and the release of tumor antigens by irradiated tumor cells. These neoantigens are taken up by antigen-presenting cells (APCs), such as dendritic cells (DCs) and phagocytic cells. The APCs interact with tumor antigens and then migrate to the lymph nodes where they present antigens to T cells, a process that is mediated by the MHC pathway and other co-stimulatory signals, such as CD80 and CD28. After activation by multiple signals, T cells, especially the CD8+ T cells, are activated and begin to propagate. As a result, activated effector T cells exit the lymph nodes and home to tumors, including primary tumors and non-irradiated tumor metastases, to exert their effect of killing tumor cells. However, cytotoxic T lymphocyte-associated antigen 4 (CTLA-4) competitively combines with CD80/86 and inhibits the activation of T cells. Following T cell activation, programmed cell death 1 (PD-1) receptors that are expressed on the T cell surface bind primarily to programmed death-ligand 1 (PD-L1) and inhibit immune responses. The administration of immune checkpoint blockades of CTLA-1, PD-1, and PD-L1 can enhance the anti-tumor immunity of RT (92).

The abscopal effect was first described for melanoma in the 1970s and has been linked to mechanisms involving the immune system (87). The abscopal effect likely also plays a role in the treatment of HCC, as demonstrated in murine models. For example, Park et al. reported that RT increased antitumor immune response and the addition of PD-L1 augmented this effect (88). RT causes cellular death and expression of tumor antigens and damage associated molecular patterns (DAMPs), which attract antigen presenting cells such as dendritic cells and actives CD8+ T-cells (89). These cells infiltrate into the tumor microenvironment and promote tumor cell death (15). RT also upregulates immune checkpoint molecules (PD-1. PD-L1, and CTLA-4), which dampens anti-tumor activity. ICI therapy is proposed to block these molecules to restore cytotoxic and anti-tumor activities of T-cells and (Anti-PD-1/PD-L1) dampen the effects of regulatory T cells (90). This proposed synergist mechanism transforms “cold” tumors with low immune cell presence to “hot” tumor with more immune cell infiltration. The use of triple therapy or combination fo PD-1/PD-L1 with RT has been demonstrated to be safe and well tolerated in patients with minimal treatment related adverse effects (91). **Figure 2** demonstrates the abscopal effect (92).

Radiation induced DNA damage activates DNA damage repair pathways that causes upregulation of CTLA-4 and PD-L1 expression causing immunosuppression within the tumor microenvironment and blunting the effects of ICI (81, 93). Ataxia telangiectasia and Rad3-related protein (ATR), a kinase in the DNA damage repair pathway, may be important in controlling immunosuppression in the tumor microenvironment. Sheng et al. assessed AZD6738, an ATR inhibitor, in a HCC murine model and demonstrated that AZD6738 increase radiotherapy stimulated CD8+T-cell infiltration and activation and reversed the immunosuppressive effects of radiation (94). As such, balancing the immune activation and suppression of RT relative to its effect on ICI is a key concept of ongoing research in the treatment of HCC. In particular, the synergistic effects of RT and ICI in the treatment of HCC works may work in several ways. For example, RT increases diffusion of immune cells into the tumor microenvironment allowing anti-tumor effects, while simultaneous anti-PD-L1, anti-PD1 and anti-CTLA-4 therapy can offset the immunosuppressive consequences induced by RT (90).

Currently, prospective clinical data on the combination RT and ICI in the treatment of HCC are lacking. A 2022 small retrospective study by Su et al. reported on 29 patients with advanced HCC (Child-Pugh A) who were treated with proton beam radiotherapy, as well as anti-PD1 or anti-PD-L1 therapy. The authors reported a median progression free survival of 27.2 months and concluded that combination therapy with RT and ICI was safe and effective for treatment of advanced HCC (95). There are other clinical trials investigating the use of RT and ICI. For example, a phase 2 trial by Tai et al. evaluated the safety and efficacy of sequential RE followed by nivolumab among patients with advanced HCC. Forty patients with unresectable HCC and Child-Pugh A cirrhosis were treated with RE with Y90, with nivolumab started 3 weeks later. The primary outcome was ORR, which was 30.6%. Serious adverse effects (14%) including Steven-Johns syndrome, hepatitis, fever, liver abscess, and ascites (96). Similarly, another phase 2 trial from 2022 assessed SBRT and camrelizumab, an anti-PD1 monoclonal antibody among patients with unresectable HCC. The study enrolled 21 patients with advanced HCC and Child-Pugh A/B liver function and reported an ORR, PFS, and OS of 52.4%, 5.8 months, and 14.2 months, respectively. No severe adverse events were noted (97). The START-FIT trial another single arm phase 2 study treated patients with advanced HCC with sequential transarterial chemoembolization (TACE) then SBRT followed by avelumab, an anti-PD-L1 monoclonal antibody. In this study, 33 patients were enrolled; 4 (12%) subsequently qualified for resection or ablation, and 14 (42%) had complete radiographic response. Adverse events included transaminitis, as well as hepatitis and dermatitis. TACE has been compared with combination RT and immunotherapy; of note, combined therapy with RT and immunotherapy has been noted to have an improved 1- and 2-year PFS and OS (98). Two recent clinical trials are focused on investigating nivolumab and RT for advanced HCC. NASIR-HCC is a single arm phase 2 trial that investigated patients with advanced HCC who underwent RE followed by nivolumab treatment; ORR was 41.5% and OS was 20.9%. A separate phase 1 trial compared SBRT plus nivolumab and ipilimumab versus SBRT and nivolumab alone. Preliminary data have suggested a favorable ORR, PFS and median OS in the SBRT plus nivolumab and ipilimumab; however the trial was stopped prematurely due to poor accural (99). A prospective study from Yu et al. demonstrated that concurrent application of RT during nivolumab treatment resulted in prolonged PFS and OS versus nivolumab alone in a cohort of 76 patient with advanced HCC (100). Smith et al. reported on combination nivolumab and upfront RT among patients with advanced HCC and demonstrated an ORR 35%, which was higher than nivolumab alone. Of note, there is an ongoing phase Ib clinical trial assessing the safety and tolerability of neoadjuvant SBRT and tislelizumab, a PD-1 inhibitor, prior to hepatic resection in patients with resectable HCC (101). There are several other ongoing clinical trials currently enrolling; a summary of these trials is provided (**Tables 1**, **2**).

**Table 1:** Recently published clinical trials using combined RT and ICI for treatment of advanced hepatocellular carcinoma.

Trial Name	Year Published	Phase	Treatment	Patient Population	Primary End Point
CA 209-678 NCT03033446	2021	II	RE + Nivolumab	Advanced HCC, Child-Pugh A	ORR
NCT04193696	2022	II	SBRT + Camrelizumab	Advanced HCC, Child-Pugh A/B	ORR and Safety
START-FIT NCT03817736	2023	II	TACE + SBRT + Avelumab	Advanced HCC, Child-Pugh A/B	Patients able to undergo curative treatment
NASIR-HCC NCT03380130	2022	II	SIRT + Nivolumab	BCLC B2 tumors	Safety, ORR, and OS
NCT03203304*	2023	I	SBRT + Nivolumab or Nivolumab + ipilimumab	Advanced HCC	Dose-limiting toxicity, ORR, PFS, OS

RE, Radioembolization; HCC, Hepatocellular Carcinoma; ORR, Objective Response Rate; SBRT, Stereotactic Body Radiation Therapy; TACE, Trans arterial Chemoembolization; SIRT, Selective Internal Radiation Therapy; OS, Overall Survival; PFS, Progression Free Survival.

*Trial stopped due to poor accrual.

**Table 2:** Ongoing clinical trials using combined RT and ICI for treatment of advanced hepatocellular carcinoma.

Trial Name	Start Date	Phase	Treatment	Patient Population	Primary End Point
NCT04167293	2019	III	SRBT + Sintilimab vs SBRT alone	Advanced HCC with portal vein invasion	PFS at 24 weeks
NCT04913480	2020	II	SBRT + Durvalumab	Advanced HCC	PFS at 1 year
NCT05488522	2022	I	SBRT + Atezolizumab + Bevacizumab	Advanced HCC	Safety and efficacy
NCT04541173	2020	II	Y90 TARE + Atezolizumab + Bevacizumab	Advanced HCC	PFS at 12 months
NCT05377034	2022	II	SIRT-Y90 + Atzezolizumab + Bevacizumab	Locally Advanced HCC	Best Overall Response Rate at 12 months
NCT05701488	2023	I	SIRT + Durvalumab + Tremelimumab	Resectable HCC	Adverse Events
NCT04169399	2019	II	SBRT + Toripalimab	Advanced HCC with portal vein invasion	PFS at 6 months
NCT04988945	2020	II	TACE + SBRT + durvalumab + tremelimumab	Advanced HCC	Downstaging to resection
NCT03857815	2019	II	SBRT + Sintilimab	Advanced HCC	PFS at 2 years
ChiCTR 210049831	2022	II	IMRT + atezolizumab + bevocizumab	Advanced HCC with portal vein tumor thrombosis	ORR
NCT05185531	2022	I	Neoadjuvant SBRT + tislelizumab	Resectable HCC	Tumor response, safety and tolerability
NCT03316872	2018	II	SBRT + Pembrolizumab	Advanced HCC	ORR

HCC, Hepatocellular Carcinoma; ORR, Objective Response Rate; SBRT, Stereotactic Body Radiation Therapy; TACE, Trans arterial Chemoembolization; TARE, Trans arterialradioembolization; SIRT, Selective Internal Radiation Therapy; OS, Overall Survival; PFS, Progression Free Survival; IMRT, Intensity-modulated Radiotherapy.

## Future directions and challenges to progress

ICI and RT in combination can be effective for the treatment of advanced HCC. The main targets for ICI, which are also impacted by RT, are PD-1/PD-L1 and CTLA-4. Combined ICI and RT may not be effective in all patients with HCC. The reasons for this may be related to the heterogeneity associated with HCC tumors and underlying etiology driving changes at the molecular level affecting the tumor microenvironment. As a result, identifying novel targets for therapy is paramount. Radiation damage to tumor cells induces apoptosis which releases numerous antigens that present potential targets for intervention. Among these are T-cell immunoglobulin mucin-3 (TIM-3), lymphocyte activation gene-3 (LAG-3), and B and T lymphocyte attenuator (BTLA). There are several ongoing clinical trials looking at inhibitors of these antigens including cobolimab (anti-TIM-3) and dostarlimab (anti-PD-1) as well as relatlimab (anti-LAG-3) (NCT03680508, NCT04567615, NCT05337137, NCT04658147) (102).

Recent study has demonstrated that erythroid progenitor cells (EPCs) in the spleen play a role in tumor progression and the subsequent immune response. The proposed mechanism involves creation of reactive oxygen species, and expression of PD-L1 leading to T-cell suppression (103). Among patients with HCC, EPCs produce artemin, a glia cell derived neurotrophic factor that stimulates HCC growth in animal models (104, 105). Combination RT and ICI therapy, particularly anti-PD-L1 could disrupt this pathway by inhibiting accumulation of splenic EPCs. Future research should focus on EPCs as a mechanism by which combination RT and ICI therapy may work.

Chimeric Antigen Receptor T Cell (CAR-T) has also been studied in the treatment of HCC. This technique takes T cells from the patient and engineers the cells to attack certain tumor cells and antigens. A number of potential antigens have been identified as targets for CAR-T therapy, including AFP, GPC-3, MAGE, NY-ESO-1, hTERT, NKG2DL, EpCAM, CD133, CD147, and MUC1 (106). As RT causes the release of innumerable tumor antigens, the combined use of RT and CAR-T therapy may hold promise for the future. Given the number of antigens released from HCC cells, biomarkers to predict which patients may benefit from certain therapies has also been an emerging researched field. Unfortunately, to date, studies have not been able to identify reliable predictive biomarkers for HCC. Markers that have been studied are PD-L1, PD-1, TIM-3, and cytolytic T cell infiltrates, as well as radiosensitive gene signatures (14, 107–110).

Further emerging areas in the treatment of HCC are use of anti-vascular endothelial growth factor (VEGF) therapy. VEGF overexpression can occur in patients with HCC and be responsible for angiogenesis and hypervascularity of HCC tumors, as well as be associated with poor prognosis (111–114). Angiogenesis has long been known to potentiate tumor formation and provides a novel target for drug therapy (115). The landmark IMbrave 150 trial demonstrated that anti-VEGF therapy increased OS and PFS when used with atezolizumab versus sorafenib (9). The mechanisms by which anti-VEGF therapy work has been studied in animal models. Mice treated with VEGF inhibitors had augmented PD-1 targets on T-cells. Combining anti-VEGF and anti-PD-1 therapy allowed for T-cells to function properly and decrease inhibitor immune cells such as Tregs (116). Radiation increases VEGF expression in HCC cells and thus RT may play a role in strengthening the effect of anti-VEGF therapy, similar to ICI therapy (114). A multicenter prospective study of 30 patients from China assessed the efficacy and safety of intensity modulated RT and systemic atezolizumab and bevacizumab in patients with HCC and extrahepatic portal vein tumor thrombus. The authors reported an ORR and median OS of 76.6% and 9.8 months, respectively, with an acceptable safety profile (117). An ongoing phase II trials is currently assessing atezolizumab and bevacizumab plus RT in patients with unresectable HCC and portal vein tumor thrombus with the results expected in the upcoming years (118). Currently, combination therapy with RT and anti-VEGF should be used with caution, however, as adverse effects with anti-VEGF after RT can be severe (119).

Using ICI and RT may help convert unresectable HCC into resectable disease allowing for R1 or R0 resection, known as conversion therapy. Although hepatectomy after conversion therapy may be more challenging, it has been proven to be safe and effective (120). Multiple studies have reported that combination therapy with RT plus either locoregional therapy such as hepatic artery infusion pump or targeted therapy can convert unresectable disease with portal vein tumor thrombus into candidates for resection (121, 122). The role of RT plus ICI in conversion therapy will be an area of active future research. Despite these exciting future directions and progress with ICI and RT, many challenges remain in the treatment of patients with advanced HCC. Most studies on ICI and RT involve Child-Pugh A patients with preserved liver function and functional status. Applying data to patients with worse liver function (i.e., Child-Pugh B/C patients) is challenging. To this point, there are relatively few studies on patients with poor liver function. The CELESTIAL trial did include patients with Child-Pugh B cirrhosis who were treated with cabozantinib, a tyrosine kinase inhibitor (123). Certain trials have advised caution, however, when using certain ICI in patients with poor liver function (124). The data have demonstrated modest efficacy and safety in select subsets of patients with advanced HCC and compromised liver function.

Another challenge with combined RT and ICI is the development of treatment resistance. HCC is a heterogenous tumor with a varied tumor microenvironment, which complex including the extracellular matrix, immune cells, cancer-associated fibroblasts, among others. This heterogeneity makes development of treatment that universally covers all tumors difficult and makes HCC relatively chemo-resistant (125–127). Elucidating mechanisms of resistance in HCC will be crucial to the development of future treatments. An additional challenge is identification of patients who will benefit most from combination therapy, as well as patient selection for combined RT and ICI. For example, SBRT combined with ICI therapy may be of benefit to patients with tumor thrombus in the portal vein, hepatic veins, or vena cava (128–130). Patients with vascular invasion may benefit from combination therapy, however further study is necessary. The timing and sequence of ICI and RT (concurrent or sequential) to maximize benefit is unclear and will also need to be defined in the future (131).

## Conclusion

Although HCC remains a leading cause of cancer death, great strides have been made in recent years in the treatment of advanced disease. Among these, ICI therapy has given the opportunity to increase survival in patients who have failed standard first line therapy. The addition of RT helps to augment the effects of ICI, although the mechanisms behind this effect continue to be studied. Despite this great progress, work remains to better identify which tumors respond best to which drugs, given the heterogeneity of HCC. Further research and progress into new drug therapy, predictive biomarkers, and mechanisms of resistance to certain drugs as well as patient selection and sequence of therapy will be crucial as we move into the next generation of treatments for this lethal disease.

## Author contributions

All authors equally contributed to the literature review, writing, and editing of this manuscript.
